# Effectiveness of isoniazid preventative therapy in reducing incidence of active tuberculosis among people living with HIV/AIDS in public health facilities of Addis Ababa, Ethiopia: a historical cohort study

**DOI:** 10.1186/s12879-016-2109-7

**Published:** 2017-01-03

**Authors:** Mahlet Semu, Teferi Gedif Fenta, Girmay Medhin, Dawit Assefa

**Affiliations:** 1 Addis Ababa Health Bureau, Addis Ababa, Ethiopia; 2 Social and Administrative Pharmacy Working Group, Department of Pharmaceutics and Social Pharmacy, College of Health Sciences, Addis Ababa University, P.O. Box 1176, Addis Ababa, Ethiopia; 3 Aklilu Lemma Institute of Pathobiology, Addis Ababa University, Addis Ababa, Ethiopia; 4 KNCV TB Care I-Ethiopia, Addis Ababa, Ethiopia

**Keywords:** IPT, Incidence of active TB, TB/HIV co-infection, Ethiopia

## Abstract

**Background:**

Human Immunodeficiency Virus (HIV) pandemic has exacerbated tuberculosis disease especially in Sub-Saharan African countries. The World Health Organization (WHO) and Joint United Nations Program on HIV/AIDS (UNAIDS) have recommended Isoniazid Preventive Therapy (IPT) for HIV infected patients to reduce the burden of tuberculosis (TB). Ethiopia has been implementing IPT since 2007. However, effectiveness of IPT in averting occurrence of active tuberculosis among HIV infected patients has not been assessed.

**Methods:**

Retrospective cohort study was employed using secondary data from public health institutions of Addis Ababa. Descriptive statistics and Generalized Linear Model based on Poisson regression was used for data analysis.

**Results:**

From 2524 HIV infected patients who were followed for 4106 Person-Years, a total of 277 incident Tuberculosis (TB) cases occurred. TB Incidence Rate was 0.21/100 Person-Year, 0.86/100 Person-Year & 7.18/100 Person-Year among IPT completed, in-completed and non-exposed patients, respectively. The adjusted Incidence Rate Ratio (aIRR) among IPT completed vs. non-exposed patients was 0.037 (95% CI, 0.016-0.072). Gender, residence area, employment status, baseline WHO stage of the disease (AIDS) and level of CD4 counts were identified as risk factors for TB incidence. The aIRR among patients who took Highly Active Anti- Retroviral Therapy (HAART) with IPT compared to those who took HAART alone was 0.063 (95% CI 0.035-0.104). IPT significantly reduced occurrence of active TB for 3 years.

**Conclusions:**

IPT significantly reduced tuberculosis incidence by 96.3% compared to IPT non-exposed patients. Moreover concomitant use of HAART with IPT has shown a significant reduction in tuberculosis incidence by 93.7% than the use of HAART alone. Since IPT significantly protected occurrence of active TB for 3 years, its implementation should be further strengthened in the country.

## Background

Tuberculosis (TB) and Human Immunodeficiency virus/Acquired Immunodeficiency Syndrome (HIV/AIDS) are major public health threat [1–3]. Among HIV infected patients, TB is the most frequent life threatening opportunistic disease, even in those receiving Highly Active Antiretroviral Therapy (HAART) and it has been shown to be a leading cause of death [2, 4, 5]. HIV infection is also the strongest risk factor for TB disease [3]. In 2011, globally there were 34 million HIV infected patients and at least one-third of these had latent TB & 1.1 million of them developed new TB infection, of these around 79% of patients were from Sub-Saharan African countries, indicating that HIV is fueling the TB epidemics in the region [5]. In Ethiopia like many of the developing countries, TB has created major burden to the health care system due to its linkage with HIV/AIDS epidemics [6]. In 2010, TB incidence in HIV-positive patients was 48 (27–76) per 100,000 population and the prevalence of TB including among HIV positives was 572(265–947) per 100,000 population [7].

Since 1998, WHO and the Joint United Nations Program on HIV/AIDS (UNAIDS) have recommended Isoniazid Preventive Therapy (IPT) as one of the key interventions in the comprehensive HIV/AIDS care strategy to reduce the burden of TB among HIV infected patients [8]. According to WHO [2], IPT given to HIV infected patients without TB disease reduces the risk of developing TB by 33–67% for up to 48 months. Regarding the concomitant use of HAART with IPT, a meta-analysis led by WHO found out that HAART reduces the individual risk of TB disease by 65%, irrespective of the CD4 cell count but recent evidence has shown that the combined use of IPT and HAART among HIV infected patients significantly reduces the incidence of TB by up to 97% [9].

In Ethiopia, IPT provision for HIV infected patients is recommended by the national TB/HIV Collaborative Activities guideline and its implementation has been started since 2007[10]. However, the effectiveness of IPT in reducing the burden of active TB among HIV infected patients relative to those who did not take or discontinued has not been assessed yet. Moreover the clinical and programmatic factors affecting the treatment outcome among those who took have not been clearly identified. Therefore, this study assessed the effectiveness of the provision of IPT for HIV infected patients in averting occurrence of TB in the Ethiopian context.

## Methods

A multi-centered retrospective cohort study design was employed using secondary data from 14 public health facilities giving ART service in Addis Ababa. From seven facilities which were giving IPT service between 2007 and January 2010, all adult HIV positive patients (1264) who were either on HAART or Pre-ART and exposed to IPT in the study period and who were followed for at least a year were included as IPT exposed patients. All IPT discontinued patients regardless of their follow up time were also included. For comparison, equivalent number of non- IPT exposed patients who were on HIV care management for 1 or more years were sampled from randomly selected facilities which were not providing IPT till January 2010. All pediatric HIV positive patients regardless of whether they took/did not take IPT in the study period set, and all transferred in patients were excluded from this study. The patient charts from each facility were selected by random sampling method. A structured data abstraction format was used to collect information from medical records/chart of patients and the data was collected from July 1 to August 31, 2012. The data was entered and processed using SPSS version 16 statistical software. Descriptive statistics for patient characterization, Generalized Linear Model based on Poisson distribution to get incidence rate and incidence rate ratio, nonparametric test and Chi Square test were used for statistical analysis.

## Results

A total of 2528 patients’ charts were reviewed and four charts were with incomplete information and hence only 2524 charts were included in analysis. Of these, 1582(62.7%) were female and their mean age was 34.9 years. Majority were married and from Orthodox family. A significant proportion (44.9%) was un-employed. There is significant difference in level of education, marital status and employment status (Table 1).

**Table 1:** Socio-demographic characteristics of patients at enrollment for chronic HIV care in public health facilities of Addis Ababa, during 2007-June 2012

Socio-demographic profile at enrollment for HIV care (*N* = 2524)	IPT Exposed *N* (%)	IPT Non-exposed *N* (%)	Total *N* (%)	Chi-square *P* value
Sex				
Male	473(37.4)	469(37.2)	942(37.3)	0.918
Female	791(62.6)	791(62.8)	1582(62.7)	
Age in years (mean = 34.9, SD = 9.1)				
<30	421(33.3)	557(44.2)	978(38.7)	0.150
30–39	408(32.3)	412(32.7)	820(32.5)	
40–49	283(22.4)	247(19.6)	530(21)	
>50	152(12)	44(3.5)	196(7.8)	
Marital status				
Single	318(25.2)	288(22.9)	606(24)	0.000
Married	584(46.2)	551(43.7)	1135(45)	
Widowed	189(15)	195(15.5)	384(15.2)	
Separated	157(12.4)	165(13.1)	322(12.8)	
Divorced	16(1.3)	61(4.8)	77(3.1)	
Level of Education				
No formal education	248(19.6)	276(21.9)	524(20.8)	0.004
Primary completed	507(40.1)	447(35.5)	954(37.8)	
Secondary completed	473(37.4)	473(37.5)	946(37.5)	
Tertiary completed	36(2.8)	64(5.1)	100(4)	
Religion				
Orthodox	979(77.5)	1023(81.2)	2002(79.3)	0.197
Muslim	98(7.8)	177(14)	275(10.9)	
Protestant	132(10.4)	54(4.3)	236(9.4)	
Others^a^	5(0.4)	6(0.5)	11(0.4)	
Employment status				
Non-employed	650(51.4)	483(38.3)	1133(44.9)	0.000
Self-employed	218(17.2)	247(19.6)	465(18.4)	
Government employed	205(16.2)	248(19.7)	453(17.95)	
Private	183(14.5)	270(21.4)	453(17.95)	
Student	8(0.63)	12(0.95)	20(0.8)	
Residence place				
Addis Ababa	1230(97.3)	1252(99.4)	2482(98.3)	0.322
Out of Addis Ababa	34(2.7)	8(0.6)	42(1.7)	
Average no. of personnel per family	3.15	3.45	3.3	0.092
Average no. of rooms per family	1.35	1.65	1.5	0.593

^a^Catholic and Joba

All of the patients were Pre-ART at the time of enrollment for chronic HIV care and the baseline clinical information of the patients is shown in Table 2. Majority of the patients were in stage 3 followed by stage 2, 972 (38.5%) and 833 (33%), respectively. There was significant clinical difference between the two groups.

**Table 2:** Baseline clinical information of HIV positive patients in public health facilities of Addis Ababa, during 2007-June 2012

Baseline Clinical status (*N* = 2524)	IPT Exposed *N* (%)	IPT Non-exposed *N* (%)	Total *N* (%)	Chi-square *P* value
Initial WHO stage of HIV/AIDS				
Stage 1	357(28.2)	215(17.1)	572(22.7)	0.000
Stage 2	487(38.5)	346(27.5)	833(33)	
Stage 3	392(31)	580(46)	972(38.5)	
Stage 4	28(2.3)	119(9.4)	147(5.8)	
Baseline CD_4_cells count/μl(mean = 230, SD = 176.9)				
≤200	570(45.1)	807(64)	1377(54.6)	0.000
>200	694(54.9)	453(36)	1147(45.4)	
Initial body weight in Kg (mean = 53.8, SD = 9.7)				
<50	304(24.1)	554(43.8)	858(34)	0.009
50–59	628(49.7)	418(33.1)	1046(41.4)	
60–69	208(16.5)	214(17)	422(16.7)	
>69	124(9.8)	74(5.9)	198(7.8)	
TB screened^a^				
Positive	0	40(3.2)	40(1.6)	
Negative	1264(100)	1220(96.8)	2484(98.4)	
On CPT				
Yes	1259(99.6)	1205(95.6)	2464(97.6)	0.000
No	5(0.4)	55(4.4)	60(2.4)	
OIs diagnosed				
None	1082(85.6)	907(71.9)	1989(78.8)	0.000
Bacterial infections	11(0.87)	164(13)	175(6.9)	
Viral infections	25(0.08)	88(6.9)	113(4.5)	
Fungal infections	38(3)	129(10.2)	167(6.6)	
Viral & bacterial infections	20(1.6)	13(1.03)	33(1.3)	
Bacterial & fungal infections	11(0.87)	8(0.63)	19(0.8)	
Fungal & viral infections	8(0.6)	12(0.95)	20(0.8)	

^a^A patient is TB positive, if he/she has at least two of these signs/symptoms: Weight loss greater than or equal to 5% of the initial weight, coughing for 2 weeks, night sweat, night-mar, Loss of appetite

As shown in Table 3, from all patients for whom their charts were reviewed, 2046 (81.1%) had initiated HAART during their follow up; of whom 945 (46.2%) of them were WHO stage 3.

**Table 3:** Clinical information of patients who were unheard follow upinpublic health facilities of Addis Ababa, during 2007-June 2012

Clinical information when ART initiated (*N* = 2046)	IPT exposed *N* (%)	IPT Non-exposed *N* (%)	*N* (%)	Chi-square *P* value
WHO stage of HIV/AIDS				
Stage 1	151(16.02)	138(12.5)	289(14.1)	0.000
Stage 2	367(39)	294(26.6)	661(32.3)	
Stage 3	394 (41.8)	551(49.9)	945(46.2)	
Stage 4	30 (3.18)	121(11.0)	151(7.4)	
CD_4_cells count/μl(mean = 151.2, SD = 84.9)				
≤200	537 (37.9)	1020(92.4)	1557(76.1)	0.000
>200	263 (27.9)	226(20.5)	489(23.9)	
Weight in Kg (mean = 53.2, SD = 9.5)				
<50	241(25.6)	506(45.8)	747(36.5)	0.011
50–59	479(44.2)	366(33.1)	845(41.3)	
60–69	164 (17.4)	164(14.9)	328(16.0)	
>69	58 (6.2)	68(6.2)	126(6.2)	
TB screened				
Yes	942(100)	1100(99.6)	2042(99.8)	0.64
No	0	4(0.4)	4(0.2)	
CPT adherence				
Good	791(84)	1096(99.3)	2032(99.6)	0.046
Fair	3(.32)	6(0.5)	9(0.3)	
Poor	3(.32)	2(0.2)	5(0.2)	
ART adherence				
Good	937(99.5)	1100(99.6)	2037(99.6)	0.124
Fair	5(0.53)	2(0.2)	7(0.3)	
Poor	0	2(0.2)	2(0.1)	
OIs after ART initiated				
None	937(99.5)	1052(95.3)	1989(97.21)	0.000
Fungal	3(.32)	20(1.8)	23(1.12)	
Viral	0	12(1.0)	12(0.5)	
Bacterial	0	12(1.0)	14(0.7)	
Bacterial & fungal	0	4(0.4)	4(0.2)	
Others^a^	0	4(0.4)	4(0.2)	

^a^Protozoal, viral & bacterial & fungal & viral infections

Out of 1264 patients who were given IPT, completion rate was 975(77.1%). Among IPT exposed patients; 942(74.5%) were on HAART and of these 738(78.3%) completed IPT (Table 4).

**Table 4:** Profile of IPT exposed patients inpublic health facilities of Addis Ababa, during 2007-June 2012

Profile (*N* = 1264)	*N* (%)	Chi-square *P*-value
Male	473(37.4%)	
Female	791(62.6%)	
TB screened & tested negative	1264(100%)	
Currently on HAART	942(74.5%)	
Pre-ART	322(25.5%)	
HAART initiated before IPT	578(61.3%)	
HAART initiated after IPT	364(38.7%)	
IPT completed	975(77.1%)	
IPT discontinued	289(22.9%)	
On HAART + IPT completed	738(78.3%)	0.094
Pre-ART + IPT completed	237(73.6%)	
On HAART + IPT in-completed	204(21.7%)	0.08
Pre-ART + IPT in-completed	85(26.4%)	
Male IPT completed	359(75.9%)	0.459
Female IPT completed	616(77.8%)	
Took B_6_ together with INH	581(46%)	
No IPT side effects	1254(99.2%)	

**Fig. 1:**
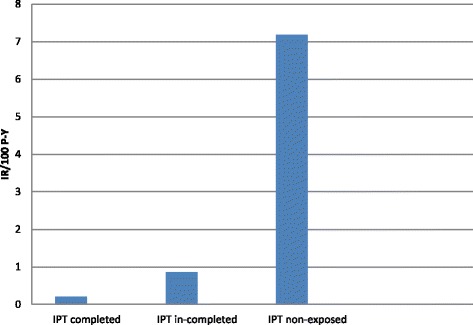
Incidence rate among IPT completed, in-completed and non- exposed patientsin public health facilities of Addis Ababa, during 2007-June 2012

Among 2524 HIV-infected patients who were followed for 4106 P-Y, 277 incident TB cases occurred, making the overall incidence of 6.7/100P-Y. Among IPT completed group, incidence rate was 0.21/100PY, while in IPT non-exposed patients; it was 7.18/100P-Y. Incidence of TB was found to be associated with sex, employment status, baseline WHO stage of HIV/AIDS and CD_4_ count. Completion of IPT showed significant protective effect against occurrence of active TB when compared to IPT non-exposed patients aIRR = 0.037 (CI 95% 0.016-0.072)} (Table 5, Fig. 1).

**Table 5:** Incidence rate, univariate and multivariate analysis among IPT completed, in-completed and non- exposed patients in public health facilities of Addis Ababa, during 2007-June 2012

Patient profile	Event/P/Y	IR/100P-Y	Crude IRR (95% CI)	Adjusted IRR(95% CI)
Over all	277/4106	6.7		
IPT completed	7/33.3	0.21	0.03(0.01–0.06)	0.04(0.05–0.07)
IPT in-completed	8/7.8	0.86	0.84(0.36–1.33)	0.89(0.49–1.76)
IPT non-exposed	262/36.49	7.18	1	1
Sex				
Male	138/2822.1	4.89	1.80(1.42–2.28)	1.59(1.20–2.12)
Female	139/5129.2	2.71	1	1
Age group in year				
<30	103/3038.3	3.39	1	1
30–39	87/2628.4	3.31	0.98(0.73–1.29)	0.82(0.61–1.11)
40–49	61/1644.2	3.71	1.09(0.79–1.49)	1.04(0.74–1.47)
>49	26/637.3	4.08	1.201(0.77–1.82)	1.55(0.95–2.44)
Residence area				
Living out of Addis Ababa	4/135.1	2.96	0.85(0.26–1.99)	0.29(0.09–0.73)
Living in Addis Ababa	273/7800	3.50	1	1
Religion				
Orthodox	231/6243.2	3.70	0.63(0.20–3.82)	0.29(0.08–1.86)
Muslim	19/892	2.13	0.36(0.11–2.28)	0.17(0.04–1.13)
Protestant	25/778.8	3.21	0.55(0.16–3.40)	0.32(0.09–2.12)
Others*	11/187.7	5.86	1	1
Marital status				
Married	127/3547.5	3.58	0.82(0.46–1.68)	1.22(0.66–2.51)
Single	72/1889.8	3.81	0.88(0.48–1.81)	1.18(0.63–2.48)
Widowed	33/1284	2.57	0.59(0.30–1.27)	0.81(0.40–1.76)
Divorced	35/997.2	3.51	0.81(0.45–1.72)	1.05(0.53–2.27)
Separated	10/229.9	4.35	1	1
Employment				
Self employed	87/3782.6	2.30	0.03(0.01–0.19)	0.02(0.01–0.12)
Private Employed	58/1418.1	4.09	0.06(0.02–0.35)	0.03(0.01–0.16)
Government employed	62/1324.8	4.68	0.07(0.02–0.40)	0.03(0.01–0.19)
Student	3/66.5	4.51	0.06(0.01–0.48)	0.01(0.002–0.101)
Non- employed	65/1354.2	4.80	1	1
Educational level				
None educated	42/1707.3	2.46	0.65(0.35–1.33)	1.25(0.62–2.70)
Primary Completed	106/3028.6	3.50	0.93(0.52–1.83)	1.69(0.89–3.49)
Secondary Completed	118/2920.8	4.04	1.07(0.60–2.10)	1.76(0.97–3.55)
Tertiary completed	11/291	3.78	1	1
House Hold size/room no.			0.99(0.89–1.09)	0.98(0.88–1.07)
Baseline WHO stage of HIV/AIDS				
Stage 1 & 2	45/4545.5	0.99	0.14(0.10–0.19)	0.23(0.16–0.31)
Stage 3 & 4	232/3381.9	6.86		1
Baseline CD_4_ cells/μl				
≤200	211/4169.9	5.06	2.89(2.21–3.85)	1.36(1.02–1.84)
>200	66/3771.4	1.75		
Baseline weight in Kg				
≤49	113/2646.4	4.27	1.88(1.12–3.43)	1.63(0.93–3.05)
50–59	108/3343.7	3.23	1.42(0.85–2.59)	1.19(0.69–2.22)
60–69	42/1333.3	3.15	1.39(0.78–2.64)	1.06(0.59–2.03)
>69	14/616.7	2.27	1	1
Baseline OIs				
Yes	194/6278.3	3.09	0.62(0.48–0.80)	1.24(0.95–1.63)
No	83/1660	5.00	1	1
CPT adherence				
Good	271/7765	3.49	0.99(0.48–2.51)	0.92(0.44–2.37)
Fair, Poor & no CPT	2/56.8	3.52	1	1

*others include: catholic, johoba and non belivers

**Fig. 2:**
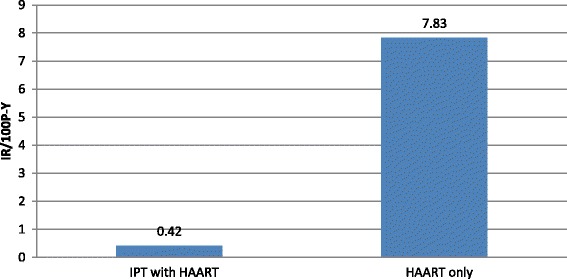
IR among patients who took HAART with IPT in public health facilities of Addis Ababa, during 2007-June 2012

Similarly, as shown in Table 6 and Fig. 2, those patients who took IPT with HAART had TB incidence rate of 0.42/100P-Y and among patients who took HAART alone, the incidence was 7.83/100P-Y. The concomitant use of IPT with HAART revealed significant protective effect on occurrence of active TB compared to HAART alone {aIRR =0.063(95% CI 0.035-0.104). Among IPT exposed patients, those who took IPT with HAART had lesser incidence than those who took IPT before initiating HAART {aIRR = 0.158(95% CI 0.039- 0.555)}.

**Table 6:** IR & IRR among patients who took HAART with or without IPT in public health facilities of Addis Ababa, during 2007-June 2012

Therapy (*N* = 1842)	IR/100P-Y	aIRR(95% CI)
IPT with HAART	0.42	0.063(0.035–0.104)
HAART only	7.83	1
IPT initiation time with HAART		
Took IPT with HAART	0.2	0.158(0.039–0.555)
Took IPT before initiating HAART	0.75	1

As shown in Table 7, there was significant CD4 and weight changes among IPT-HAART treated patients.

**Table 7:** Statistical analysis of clinical data among patients who took HAART with IPT in public health facilities of Addis Ababa, during 2007-June 2012

Variables		*N*	Mean Rank	*P*- value
Weight after completing IPT – weight at enrollment	Negative Ranks	160^a^	410.64	
	Positive Ranks	701^b^	435.65	0.000
	Ties	114^c^		
	Total	975		
CD4 after completing IPT - CD4 at enrollment	Negative Ranks	253^d^	546.73	
	Positive Ranks	702^e^	453.23	0.000
	Ties	21^f^		
	Total	976		

^a^Weight when completing IPT < weight when starting IPT

^b^Weight when completing IPT > weight when starting IPT

^c^Weight when completing IPT = weight when starting IPT

^d^CD4 when completing IPT < CD4 when starting IPT

^e^CD4 when completing IPT > CD4 when starting IPT

^f^CD4 when completing IPT = CD4 when starting IPT

The median duration of follow- up was 40 months (inter-quartile range, 28–52 months). From those who completed IPT, active TB has occurred between 6 and 28 months duration. Almost 50% of patients who completed IPT developed active TB at 19^th^month; while in non-exposed patients TB occurred within a month time of enrollment (Table 8).

**Table 8:** Month of occurrence of active TB among IPT completed, in-completed & non-exposed patients in public health facilities of Addis Ababa, during 2007-June 2012

Variables	No of patients active TB diagnosed	Minimum month TB occurred	Maximum month TB occurred	Mean month TB occurred	Standard Deviation	Median
Month TB occurred among IPT completed patients	7	6	28	16.7	8.4	19
Month TB occurred among patients who discontinued IPT	8	0	35	9.6	14.8	0
Month TB occurred among IPT non-exposed	262	0	65	8.1	13.3	1

As shown in Table 9, IPT completers were significantly protected for 3 years {aIRR = 0.04 (95% CI (0.02-1.74)} compared to IPT in-completed and non-exposed patients.

**Table 9:** TB incidence in month interval among IPT completed, in-completed & non-exposed patients in public health facilities of Addis Ababa, during 2007-June 2012

Follow up month	IPT exposure/completion status	IR/100 P-M (95% CI)	Unadjusted IRR (95% CI)	Adjusted IRR (95% CI)
<6	Completed	0	0	0
	In-complete	142.8(17.64–268.07)	1.82(0.65–3.97)	2.125(0.712–5.103)
	Non-exposed	78.59(66.9–90.3)	1	1
6–12	Completed	12(1.58–25.58)	0.30(0.06–0.79)	0.03(0.013–1.95)
	In-completed	38.71(7.74–69.68)	0.98(0.39–2.02)	1.05(0.32–2.89)
	Non-exposed	39.43(34.05–44.82)	1	1
12–24	Completed	6.74(1.35–12.14)	0.29(0.11–0.59)	0.03(0.01–0.74)
	In-completed	38.71(7.74–69.68)	1.65(0.65–3.38)	1.61(0.51–4.29)
	Non-exposed	23.51(20.48–26.55)	1	1
24–36	Completed	5.98(1.55–10.42)	0.34(0.14–0.66)	0.04(0.02–1.74)
	In-completed	9.94(3.05–16.82)	0.56(0.25–1.05)	1.09(0.49–2.54)
	Non-exposed	17.87(15.63–20.11)	1	1
37–48	Completed	5.98(1.55–10.42)	0.44(0.186–0.854)	0.74(0.301–1.56)
	In-completed	9.94(3.05–16.82)	0.73(0.33–1.37)	0.72(0.314–1.44)
	Non-exposed	13.72(12.04–19.56	1	1
>48	Completed	5.98(1.55–10.42)	0.50(0.22–0.99)	0.88(0.36–1.84)
	In-completed	9.94(3.05–16.82)	0.84(0.38–1.58)	0.89(0.39–1.76)
	Non-exposed	11.88(10.44–13.32)	1	1

## Discussion

This retrospective cohort study covering the time from 2007 to June 2012 attempted to assess effectiveness of IPT against active TB in HIV positive adults who were on HIV care in government health facilities of Addis Ababa. Accordingly, the IRs were 0.21/100P-Y, 0.86/100P-Y & 7.18/100P-Y among IPT completed, in-completed & non-exposed patients, respectively. The IR among IPT completed patients was lower when compared with the findings of studies done in different countries [11–13]. Moreover, completion of IPT in HIV infected adults significantly reduced TB incidence by 96.3% when compared to non-exposed patients (aIRR = 0.037, 95% CI 0.016-0.072). The present study revealed abetter reduction in TB incidence after IPT in comparison with the results of earlier studies [7, 14–16]. Better patient adherence rate, difference in TB burden among the countries or better socioeconomic and clinical status of patients might have contributed to such differences among studies conducted in different countries. Despite the fact that Ethiopia is among high TB burden country, IR among IPT completed patients was comparably lower than studies indicating further the effectiveness of IPT for HIV infected patients. Hence more widespread provision of IPT has the potential to further reduce TB incidence and hence improve quality of life among HIV infected adults in the country.

Among patients who completed IPT, though TB had occurred after 6 months, almost 50% of them developed TB at 19^th^month; while in IPT non-exposed patients, half of patients developed active TB within a month time. The study proved that IPT has been significantly protecting early occurrence of TB during the first 6 months. This finding was comparable to the study done in Thailand where IR among IPT completers was 0 and among non-exposed patients 8.60/100 P-Y [17]. Moreover, the present study indicated that IPT had offered a significant protective effect until 3 years. The durability of protective effect of IPT documented in the present study concurs with the expected level indicated in Ethiopian guideline [10]. It is, however, better than reports from South East Asian and other Sub-Saharan African countries [13, 17, 18].

With regard to risk factors associated with TB occurrence, even though there is significant difference among the two groups in socio-demographic and baseline clinical characteristics, the multivariable analysis revealed significant influence of IPT completion, male sex, employment status, baseline WHO stage of HIV/AIDS (stage 3 & 4) & CD_4_ cell count (less than 200cells/μl).

Similarly, South African and Namibian studies indicated the influence of clinical factors on the incidence of Tb among HIV patients [13, 16].

Getahun et al. [19] reported that in countries with a high prevalence of HIV, more women than men are diagnosed with TB. But the current study revealed that more males who took IPT were at risk of developing active TB than females (aIRR = 1.596(95% CI = 1.203-2.117). This was in agreement with the report made by Golub et al. [20].

Concerning IPT initiation time with HAART, among IPT exposed patients; those patients who took IPT with HAART had 84.2% incidence reduction than those who took IPT prior to initiating HAART. This study also noted that there was significant CD_4_cell count and weight changes after taking IPT-HAART combination therapy compared to cell count at enrollment, which might have contributed for preventing TB recurrence. This finding could serve as a base for further studies to reach an understanding on whether concomitant initiation of IPT with HAART or delayed initiation of IPT is better in terms of efficacy, toxicity or the development of immune reconstitution. In general the current finding encourages further implementation of the therapy in the country so as to decrease the burden of TB among HIV infected patients.

## Conclusions

Completion of IPT significantly reduced TB incidence by 96.3% and IPT had significantly protected occurrence of active TB for three years among HIV infected patients. Male sex, CD_4_ cell count less than 200cells/μl, WHO stage of HIV/AIDS 3 & 4 and being non-employed were risk factors for TB incidence. Concomitant use of HAART with IPT significantly decreased TB incidence by 93.7% more than HAART alone. This result evidenced and supported the WHO recommendations that IPT protected the occurrence of active TB and proved the effectiveness of IPT in reducing TB incidence among HIV infected patients in Ethiopia. Therefore, it should be implemented widely for HIV-infected patients in all parts of the country so as to improve their quality of life by reducing the TB burden and prevent further transmission of TB in the community.

## Abbreviations

IPT: Isoniazid preventive therapy
aIRR: Adjusted incidence rate ratio
HAART: Highly active antiretroviral therapy
IR: Incidence ratio
PMTCT: Prevention of mothers to child transmission of HIV
TB: Tuberculosis
WHO: World Health Organization
UNAIDS: United Nation programs on HIV/AIDS
